# Increasing Role of Targeted Immunotherapies in the Treatment of AML

**DOI:** 10.3390/ijms23063304

**Published:** 2022-03-18

**Authors:** Jochen Greiner, Marlies Götz, Verena Wais

**Affiliations:** 1 Department of Internal Medicine, Diakonie Hospital Stuttgart, 70176 Stuttgart, Germany; 2 Department of Internal Medicine III, University of Ulm, 89081 Ulm, Germany; marlies.goetz@alumni.uni-ulm.de (M.G.); verena.wais@uniklinik-ulm.de (V.W.)

**Keywords:** acute myeloid leukemia, immunotherapy, leukemia-associated antigens, molecular mechanisms, target structures

## Abstract

Acute myeloid leukemia (AML) is the most common acute leukemia in adults. The standard of care in medically and physically fit patients is intensive induction therapy. The majority of these intensively treated patients achieve a complete remission. However, a high number of these patients will experience relapse. In patients older than 60 years, the results are even worse. Therefore, new therapeutic approaches are desperately needed. One promising approach in high-risk leukemia to prevent relapse is the induction of the immune system simultaneously or after reduction of the initial tumor burden. Different immunotherapeutic approaches such as allogenic stem cell transplantation or donor lymphocyte infusions are already standard therapies, but other options for AML treatment are in the pipeline. Moreover, the therapeutic landscape in AML is rapidly changing, and in the last years, a number of immunogenic targets structures eligible for specific therapy, risk assessment or evaluation of disease course were determined. For example, leukemia-associated antigens (LAA) showed to be critical as biomarkers of disease state and survival, as well as markers of minimal residual disease (MRD). Yet many mechanisms and properties are still insufficiently understood, which also represents a great potential for this form of therapy. Therefore, targeted therapy as immunotherapy could turn into an efficient tool to clear residual disease, improve the outcome of AML patients and reduce the relapse risk. In this review, established but also emerging immunotherapeutic approaches for AML patients will be discussed.

## 1. Introduction

Even though new treatment strategies have been developed in the past years, acute myeloid leukemia (AML) still has a poor prognosis, especially in older or frail patients [1,2]. Despite intensive treatment, a high number of patients relapse, and the median overall survival for AML patients remains low [2,3]. However, the efficacy of immunotherapies in cancer treatment becomes more and more apparent. Recently, especially with immune-checkpoint inhibitors [4,5,6,7], clinical treatment algorithms of malignant diseases such as malignant melanoma, lung cancer as well as lymphoma have changed. Therefore, not only in solid tumors, but also in different haematological malignancies, the immunological approaches are becoming increasingly important. Yet many mechanisms of efficacy are still poorly understood, which is also a major potential of this form of therapy. To increase the efficacy of immunotherapeutic approaches in AML treatment, the different mechanisms must be further elucidated. Routinely used immunotherapies are allogeneic hematopoietic stem cell transplantation (alloHSCT) and donor lymphocyte infusion (DLI). The immunogenic mechanism is based on the graft-versus-leukemia (GvL) effect, in which allogeneic T cells recognize target antigens on malignant cells. While cellular approaches are already part of routine clinical practice in the treatment of AML [8,9], further immunotherapeutic approaches might be an option to prevent disease relapse.

Though there is a broad spectrum of immunotherapies that need to find their place in daily routines, including a variety of therapeutic options such as monoclonal antibodies, cytokines and immunomodulatory agents, as well as cellular immunotherapies such as vaccinations, dendritic cell treatment, and T-cell activating antibodies such as the immune checkpoint inhibitors, bispecific antibodies [10] and chimeric antigen receptor modified T cells (CARs). Additionally, different molecular targets such as FMS-like tyrosine kinase 3 (FLT3), Nucleophosmin 1 (NPM1), CCAAT/enhancer binding protein alpha (CEBPA), cKIT and DNA methyltransferases 3A (DNMT3A) and leukemia-associated antigens, such as Preferentially Expressed Antigen in Melanoma (PRAME), Survivin, Receptor for Hyaluronic Acid Mediated Motility (RHAMM), G250, Synovial Sarcoma X breakpoint 2 Interacting Protein (SSX2IP) and Wilms’ Tumor 1 (WT1), offer new strategies. Therefore, mentioned without any claim to be exhaustive, several new drugs have been promoted such as first and second generation FLT3 inhibitors, isocitrate dehydrogenase 1 and 2 (IDH1/2) -inhibitors, demethylating agents, liposomal cytarabine and daunorubicin (CPX-351), venetoclax and the hedgehog pathway inhibitor Glasdegib [11].

New immunological possibilities of treatment have to be discovered and implemented into the current treatment schedule. We discuss some of these approaches in more detail below.

## 2. Immunotherapies Targeting Immunogenic Leukemia-Associated Antigens

### 2.1. Description of Leukemia-Associated Antigens (LAAs)

Several immunogenic antigens have been identified and characterized by our group and others in a number of malignancies, including various hematological diseases, as AML [11,12,13,14]. The expression of LAA and co-stimulatory molecules in tumor cells as possible targets and how to enhance the induction of specific immune responses against these tumor cells were investigated.

Importantly, some of these target structures play a dual role, firstly in the activation of the immune system and secondly in terms of molecular mechanisms [15].

For example, patients have a more favorable prognosis if one or more LAAs such as PRAME, Proteinase 3, RHAMM, WT1 and SSX2IP are expressed in patient cells [16]. These immunogenic target structures are recognized by CD8+ T cells and can lyse tumor cells. Some of them additionally trigger a humoral immune response.

The LAA **PRAME** is a dominant repressor of the RAR signaling pathway. It binds to the retinoic acid receptor (RAR) in the presence of retinoic acid, thereby suppressing ligand-dependent receptor activation and gene transcription. By binding and thus simultaneously activating the RAR, retinoic acid normally induces transcription, resulting in differentiation processes, apoptosis or cell cycle arrest of the corresponding cells. Loss of responsiveness to retinoic acid is therefore beneficial to tumor cells. The gene is located on the human chromosomal region 22q11, encodes a 509 amino acid (AS) protein and is recognized by the HLA-A24 receptor and subsequently presented to cytotoxic T cells (CTLs). The antigen is expressed in some healthy tissue; however, levels of expression in tumor tissue are highly increased, making the antigen an interesting target of immunotherapy [17,18].

It was also found that very high and very low levels of PRAME expression correlated with poor overall survival. Higher PRAME expression could indicate a higher tumor burden and the presence of more aberrant leukemia cells, while low PRAME expression could reflect a situation where leukemia cells can escape immune surveillance [19].

***Proteinase 3*** is a protein coding gene, and a related pathway is cytokine signaling in the immune system. Quazilbash et al. used the peptide PR1, an HLA-A2-restricted peptide derived from both proteinase 3 and neutrophil elastase, which is recognized on myeloid leukemia cells by cytotoxic T lymphocytes (CTLs) that preferentially kill leukemia and contribute to cytogenetic remission in a phase I/II vaccination trial (NCT00004918) [20].

The ***RHAMM*** gene on chromosome 5q34 is particularly important in the areas of cell motility, differentiation and proliferation. It has a dual function in inflammation and tumorigenesis, particularly when interacting with CD44 in inflammatory responses and tumor development and/or progression [21].

RHAMM is normally located in the cytoplasm but can be transported to the cell surface by specific stimuli or malignant transformations through the redistribution of intracellular proteins [22,23]. High expression is also found in the healthy tissue of the testis, placenta and thymus. Very low rates were detected for pancreas and lung tissue. On CD34+ cells, surface markers for progenitor and stem cells of the blood and bone marrow, the protein is not expressed, suggesting that vaccine therapy would not affect normal hematopoiesis [24].

The HLA-A2-restricted RHAMM-R3 peptide (ILSLELMKL) is an interesting epitope to trigger a specific immune response of CD8+ effector T cells. RHAMM simultaneously induces a humoral and cellular immune response in AML patients [25].

It has already been established for a long time that the ***Wilms tumor 1 (WT1)*** gene encodes for a transcription factor that contains four zinc finger motifs at the C-terminal and a DNA-binding domain rich with proline–glutamine at the N-terminal. WT1 is a key regulatory molecule involved in the regulation of proliferation, differentiation and cell growth and survival [26]. Interestingly, it is both overexpressed and mutated in various forms of acute myeloid leukemia. It has been shown that overexpression plays a prognostic role in this disease. WT1 interacts with a variety of proteins, including p53, which is stabilized by WT1 and plays a role in preventing apoptosis. WT1 also binds to the chaperone heat shock protein 90, leading to the stabilization of WT1, and to STAT3, thus in turn leading to the increased cell proliferation of Wilms tumor cells. New evidence suggests a novel role for WT1 mutations in deregulating epigenetic programs in leukemic cells through its interaction with epigenetic modifiers TET2 and TET3 in AML [27].

Many studies have shown that WT1 is abnormally expressed or mutated in hematopoietic malignancies, including AML. Thus, it has a potential function as an MRD marker and as a possible therapeutic target [28]. Several trials of WT1 peptide vaccination have already been undertaken; results were reviewed by Di Stasi et al. [29].

**Survivin** is coded by the *baculoviral IAP repeat-containing 5 (BIRC5)* gene. It has been found to be involved in several central pathways that control cell viability and proliferation [30,31].

Survivin is a modulator of apoptotic and non-apoptotic cell death; it is involved in spindle formation and anti-apoptosis. While in normal differentiated adult tissues little or no expression of Survivin is found, high expression has been described in a number of hematological malignancies [32]. One strategy could consist of using a repressor of Survivin to block this antigen, such as has been attempted in cancer patients [33].

**Melanoma antigens (MAGEs)** not only drive tumorigenesis but also regulate pathways essential for diverse cellular and developmental processes. MAGEs are involved in molecular mechanisms in germ cell and neural development as well as oncogenic functions in cancer, and they have potential as therapeutic targets in disease [34,35].

MAGE antigens belong to the category of cancer testis antigens. MAGE itself has not been expressed at appreciable levels in AML, so it is likely that immunotherapy will need to be used in combination with other treatments, e.g., hypomethylating agents [36], which have been shown to re-express MAGE in AML blasts, or recently in a phase II clinical trial of AML patients treated with azacitidine and vorinostat, resulting in increased expression of MAGE and AML cells that can then be recognized by circulating T cells [37].

**Figure 1:**
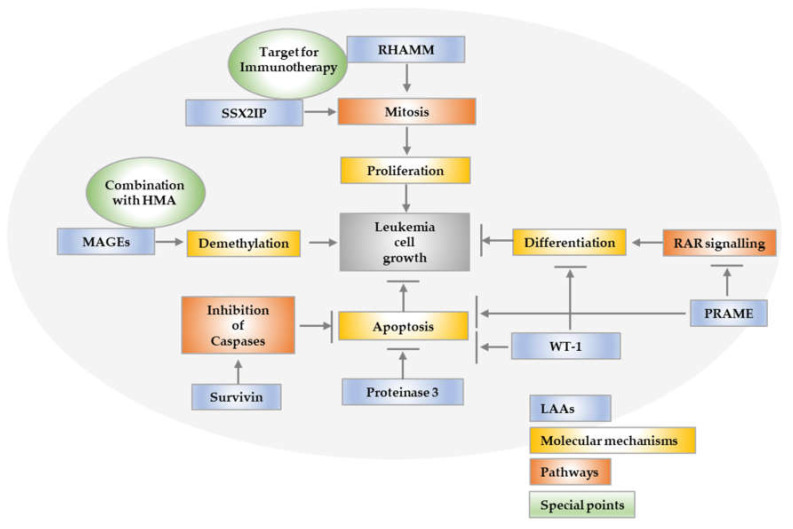
Signaling pathways and molecular mechanisms of LAA.

**SSX2IP**, a member of a family of *SSX* genes, which are only expressed in the testis and at very low levels in the thyroids of normal individuals, has been shown to peak on the surface of myeloid leukemia cells during mitosis; thus, it is involved in the control of cell proliferation (Figure 1). It was one of the first tumor antigens used as a biomarker for improved overall survival, since an elevated expression of the SSX2IP predicts survival in acute myeloid leukemia patients who lack detectable cytogenetic rearrangements. Since SSX2IP is expressed mainly on AML blast in the process of proliferation, it may be suitable to play a dual role as a predictor of overall survival and also as target for immunotherapy in leukemia [38,39].

### 2.2. Immunotherapies Targeting LAAs

There is quite a variety of vaccination strategies, which have been performed as an innovative concept in the therapy of AML. The approach is to activate the immune system against tumor antigens by peptide, DNA or RNA vaccinations [40,41]. This section describes various immunotherapies that target LAAs.

An improved clinical and immunological response after peptide vaccination with the RHAMM-R3 peptide was demonstrated in a phase I/II study. RHAMM-R3 peptide vaccination strategies in patients with malignant myeloid diseases were shown to be safe, and specific immune responses could be detected at a high frequency [42,43].

WT1 seems to be a promising antigen for eliciting a T-cell specific response [40]. One study, conducted by Keilholz et al., investigated the immunogenicity of WT1 peptide vaccination in WT1 expressing AML and MDS patients who had had no curative treatment option. Seventeen AML patients received several vaccination regimens, and this study provided insight on potential clinical efficacy in AML patients [44].

In various studies, vaccines based on WT1 proteins showed to be safe, and some patients achieved a sustained remission [29]. A combination of the vaccine (DEC-205/NY-ESO-1 fusion protein CDX-1401) and Poly ICLC, as an immunostimulant, in addition with decitabine and nivolumab, was tested in a phase I trial (NCT03358719) [40]. In another phase II trial, a vaccine based on WT1 peptide (galinpepimut-S) was used in twelve repeated dosages over approximately eleven months [40,45]. The vaccine was well tolerated, and the median disease-free survival was 17 months [40]. OCV-501 is another vaccine in a phase II clinical trial based on a helper peptide derived from the WT1 protein and restricted to HLA class II [40].

Another possibility is the development of DNA vaccines incorporating the entire sequence of an antigen. Based on DNA vaccines, an efficient T cell response to a wide range of MHC class I and II epitopes can be induced. As with the peptide-based WT1 vaccines, there is also the possibility of using WT1 as an immunotherapeutic agent with DNA-based vaccines. In preclinical experiments, mice were vaccinated with WT1 plasmid DNA, encoding the full-length murine WT1. A T cell response against the WT1 protein was evoked, and WT1-expressing tumor cells were killed. These results and numerous clinical trials as well as animal models demonstrate the ability of leukemia vaccines to evoke an immune response [20,29,45]. However, leukemic cells produce a weak immunogenicity that reduces the possible anti-leukemia effect. Therefore, isolated peptide vaccinations may be deficient to generate prolonged immunity; thus, a more complex immunotherapeutic approach might be considered [46].

A Proteinase 3 vaccination study was conducted with the peptide PR1; it involved 42 AML patients, who formed the largest group [20]. The results suggest that specific immunity may be induced in association with reduced disease activity. In addition, PR1 peptide vaccination may contribute to molecular remission, but not in advanced disease. The authors state that future studies may reveal that peptide vaccines in combination with targeted therapies might have an additional effect in reducing the leukemia burden [20].

About **Dendritic cell therapies** there is a wide range of scientific insight targeting LAAs. This is another concept to trigger an immune response based on the presentation of antigens on dendritic cells (DCs) [40,47]. DCs play a very important role in the recognition, elimination and tolerance of cancer cells. Due to this capacity, several DC-based anti-tumor vaccines have been tested in clinical trials [47]. DC-based immunotherapy has the potential to bring about clinical responses in patients with AML, particularly in post-remission settings where treatment with DCs can produce durable remissions and prevent or delay relapses in some high-risk patients, for example, combining DC vaccination with conventional therapies, as both are potentially synergistic. In a phase II clinical trial of WT1 mRNA-electroporated DC vaccination, unexpectedly high second remission rates and OS times to subsequent salvage treatment were observed in vaccinated patients that experienced the first relapse. This may indicate that DC vaccination can potentiate the response to subsequent treatment [48,49].

**TCR-modified T cells** are a new strategy targeting LAAs and also other target structures. After alloHSCT, donor T cells can mediate the beneficial GvL effect. Nevertheless, donor T cells can also be one of the main causes of morbidity and mortality after alloHSCT, namely graft versus host disease (GvHD). A favorable approach could be adoptive immunotherapy with ex vivo expanded tumor-specific T cells. A preferential target could be PRAME, which is overexpressed in several cancers including AML [50]. Ex vivo augmented PRAME-specific T cells established antigen specificity. In further analysis, the predominance of Th1 phenotype was shown, which is associated with beneficial in vivo function and prolonged persistence [50].

Another concept is HLA-DPB1-targeted CD4 T cell clones. HLA-DPB1 mismatch in alloHSCT is associated with a lower risk of leukemia relapse. Based on this concept, HLA-DPB1-targeted CD4 T-cell clones have been developed [51]. These CD4 T cells were able to eliminate leukemia blasts in AML mouse models. In conclusion, allo-HLA-DPB1-specific TCRs might be a compelling approach in the prevention of relapse after alloHSCT [51]. Thus, the genetic modification of TCR to enhance anti-leukemic activity as well as the variable expression level of HLA-DPB1 in non-hematopoietic tissues affect also non-hematopoietic cells. Considering this aspect, the hematopoiesis-restricted specificity and safety of this approach have to be improved [51,52].

### 2.3. Mutation Specific LAAs and Their Therapeutic Potential

One of the most frequently identified mutation in AML (~30%) is the **FMS-like tyrosine kinase 3 (*FLT3*)** mutation [53,54]. There are two types of *FLT3* mutations: the internal tandem duplications (ITDs), representing the most prevalent *FLT3* mutations with a frequency of 10–25%, and the single-nucleotide variants in the tyrosine kinase domain (TKDs), in 5–10% of patients [53,54]. Patients with the *FLT3*-ITD mutation have a high tumor burden. The prognosis is additionally associated with concurrent Nucleophosmin 1 (*NPM1*) mutations, the allelic ratio low (<0.05) versus high (≥0.5)) and the locations of the ITD insertion site [55]. In the RATIFY trial (ClinicalTrials.gov: NCT00651261; CALGB 10603), it was demonstrated that *NPM1* mutations or Core-Binding-Factor (CBF) rearrangements identify favorable prognostic groups in patients with FLT3-TKD [56]. Most *FLT3*-ITDs affect a single protein domain. In the search for coding immunogenic peptides of HLA class I, one of the peptides (YVDFREYEYY) induced an autologous T-cell response in vitro [57]. These peptide-reactive T cells recognized *FLT3*-ITD-mutated AML cells. This mechanism might be a possibility to eliminate MRD in *FLT3*-ITD-mutated AML [57].

Jetani et al. engineered CD8^+^ and CD4^+^ T cells expressing an *FLT3*-specific chimeric antigen receptor (CAR) and demonstrated strong reactivity against AML cells that express either wild-type FLT3 or *FLT3* with internal tandem duplication (*FLT3*-ITD). The data suggest that *FLT3*-ITD+ AML cases are particularly vulnerable and have a high likelihood of benefiting from FLT3-CAR-T cell therapy [58].

Another new approach for immunotherapy targeting FLT3 is an Fc-optimized FLT3 antibody that induces NK cell reactivity against B-ALL. Based on this finding, an interventional phase I/II study on an Fc-optimized FLT3 antibody termed 4G8-SDIE (FLYSYN) was tested in patients with AML(NCT02789254) [59]. The recruitment phase of this trial is completed; further data still needs to be collected.

***NPM1***-mutated AMLs exhibit characteristic stem cell-like gene expression profiles [60]. *NPM1* mutations are found in 15–30% of the patients, and the frequency decreases with older age [53]. *NPM-* mutated AMLs are responsive to different cytotoxic agents but also demethylating agents and new drugs such as BCL-2 inhibition [61]. Therefore, *NPM1* mutant/*FLT3*-ITDlow and *NPM1* mutant/FLT3-ITD wild-type AMLs are associated with favorable risk. However, *NPM1* mutant/*FLT3*-ITD high (allelic ratio 0.5 or greater) AML subgroups are associated with intermediate-risk disease and were mostly transplanted [53].

The transcript level of *NPM1* can be monitored by highly sensitive quantitative reverse transcription-PCR assays as MRD and is therefore used in the prognostic assessment and evaluation of the treatment course [53,62,63]. In the case of detectable MRD, there is a chance to prevent a hematological relapse early on [64]. There are different new approaches to preventing relapse in this early stage. One approach is to use NPM1 as an immunotherapeutic target [65,66,67]. In *NPM1*mut AMLs, mutation-specific peptides have been described as immunogenic target structures [68]. *NPM1*mut-specific CD4 and CD8 CTLs may be involved in the rejection of *NPM1*mut myeloid leukemia blasts [64,65]. The activation of T cells might be a strategy sufficient for maintenance in *NPM1*mut AML. In conclusion, NPM1 could trigger an immune response that could be partly responsible for the favorable prognosis of *NPM1*mut AML [65,66,67]. Another approach is Selinexor, which is an exportin 1(XPO1) inhibitor [69]. Prospective randomized trials are ongoing.

## 3. Immunotherapeutic Strategies Targeting Cell Surface Structures

### Antibody-Directed Immunotherapies

**Gemtuzumab-Ozogamicin (GO)** is an antibody-drug conjugate that comprises a CD33-antibody with a cytotoxic derivative of calicheamicin [70]. GO is approved in the treatment of CD33-positive AML in combination with chemotherapy. However, in initial phase III trials, the data were inconsistent [71]. Due to increased deaths in patients treated with GO and intensive chemotherapy, in 2010, GO was actually withdrawn from the market [70]. As a result, a further phase III trial (EudraCT 2007-002933-36) was conducted [71]. In this clinical trial, low-dose fractioned GO was administered in combination with standard chemotherapy [71]. Due to this approach, the cumulative GO doses were higher, and the toxicity and survival outcome in patients with favorable and intermediate cytogenetic-risk disease was significantly improved [70,71]. In the AMLSG 09-09 trial for *NPM1*mut AML patients, event-free survival and cumulative incidence of relapse was favorably influenced in females, patients ≤ 70 years and FLT3-ITD negative AML [72]. The combination of GO with intensive chemotherapy caused a higher clearance of *NPM1*mut transcript level, which resulted in a lower relapse rate [71,73].

**Tagraxofusp-erzs (SL-401)** is an anti-CD123 drug-conjugated antibody (IMGN632) [74]. It was approved in 2018 for patients with blastic plasmacytoid dendritic cell neoplasm (BPDCN). Up to date, there are studies for Tagraxofusp in AML and other hematological malignancies (NCT04342962). More drugs targeting CD123 are under investigation and construction [74].

In older or unfit AML patients, hypomethylating agents (HMA) now often in combination are the standard of care [75]. However, response rates are modest and rather short. Under treatment with HMA, leukemic stem cells (LSC) upregulate CD70. In this setting, **Cusatuzumab**, a human CD70 monoclonal antibody, showed in phase I/II trials substantial LSC reduction. There were no dose-limiting toxicities reported (NCT03030612) [75]; therefore, it might be an interesting approach. However, up-to-date ongoing trials are active, but not recruiting.

Another antibody directed tool is the treatment with anti-CD47 antibodies. CD47 is expressed on cancer cells and inhibits the phagocytosis via interaction with SIRP-α on macrophages [76]. Enhanced CD47 expression on cancer cells is associated with poor overall survival. **Magrolimab** as an anti-CD47 antibody interferes with this escape mechanism, which results in natural immune activation against cancer cells. In clinical trials, Magrolimab has shown efficacy in high-risk myelodysplastic syndrome and TP53 mutant AML [76]. In an ongoing phase Ib/II study, Magrolimab is tested in combination with venetoclax and azacitidine in relapsed and refractory AML. First promising results with high CR/CRi (94%) rates were shown at ASH 2021 (NCT04435691; [77]). Further pivotal phase III studies are ongoing in TP5 mutant AML (NCT04778397; [78]).

**CAR-T cells in AML** may be an approach for the potentially curative treatment of AML and the prevention of relapse in the field of adoptive T-cell therapy; however, there are some important challenges that need to be overcome [79]. CD19-targeted chimeric antigen receptor (CAR) T cells have revolutionized the treatment of relapsed acute lymphoblastic leukemia, aggressive lymphoma and multiple myeloma [79,80,81,82,83]. Efforts are therefore being made to transfer this approach to AML treatment. A dilemma is to find a suitable target structure on myeloid cells because possible antigens are often co-expressed on normal hematopoietic stem cells. Furthermore, AML is a heterogenous disease with a high potential for diverse immune escape mechanisms [79]. Up-to-date CAR-T therapy for AML is already being tested in clinical trials with promising results. In a phase I trial CD123-CAR-T cells, are administered in relapsed and refractory AML. The activity of the CAR-T was demonstrated by a cytokine release syndrome (CRS). However, further aspects such as efficacy and impact on survival and will be addressed in the ongoing trial [84].

## 4. Immunotherapies with Multiple and Unknown Antigen Structures

**Allogeneic stem cell transplantation (alloHSCT) and donor lymphocyte infusion (DLI)** in the treatment of AML. That alloHSCT plays an important role in the curative treatment of leukemias has been repeatedly demonstrated [53,85]. The antileukemic response of the transplanted and newly acquired immune system by alloHSCT depends on the GvL effect [85]. Regardless of the improvement according to treatment-related mortality, relapse remains one of the main causes for treatment failure after alloHSCT [86]. Therefore, a therapeutic option to enhance the GvL effect after alloHSCT without triggering GvHD is an exquisite goal. A routinely used approach to prevent relapse as well as treat relapse after alloHSCT is the administration of unmanipulated T cells [85,86]. T cell responses against malignant cells play a major role in maintaining remission and prolonging overall survival in patients after alloHSCT and DLI due to GvL. Nonetheless, due to DLI, the risk of severe GvHD as well as serious infections might increase [85]. In summary, a better understanding of the underlying effects of DLI on immunogenic antigen recognition is needed [85]. For a better characterization of the T cell responses, Hofmann et al. assessed the frequency and diversity of leukemia-associated antigen (LAA)-specific cytotoxic T cells using ELISpot and pMHC multimer assays and analyzed the frequency of regulatory T cells (Treg) as well as cytokine profiles before/after DLI. The data were correlated to the clinical course of patients. After alloHSCT and DLI, an increase in specific CTL reactions against various LAAs was observed. Findings suggest that a broader LAA epitope-specific T cell response as well as a decreasing number of Tregs contribute to the clinical outcome of patients treated with DLI [87]. In addition, it has been shown that GvHD occurs mainly in tissues containing a large number of antigen-presenting cells such as DCs [86]. In inflamed tissue, DCs will process and present more antigens from the damaged organ, which is likely to trigger GvHD in the process. An easy solution might to administer DLI at a time and circumstance without, or with as little as possible, inflammation [86]. Especially in the transplant setting, there is a lot of inflamed tissue; therefore, an approach to maximize the GvL- while minimizing the GvHD-effect might be in vitro T-cell depletion or rather in vivo T-cell depletion due to antibodies or post Cyclophosphamide treatment [86].

An additional option for patients with AML might be working with LAA as a target. The expression of different LAAs on malignant cells is a prerequisite for specific immune responses against these LAA. [88]. Promising candidates such as WT1, PR3, hTERT, Survivin, PRAME and RHAMM were described earlier in this review [89]. However, the frequency of LAA-specific CTLs is rather low [89]. To increase the amount and activity of LAA-specific CTLs, different approaches are under investigation, for example vaccination (see vaccination section) [89]. A delicate option would be DC or peptide vaccination in combination with DLI (vaccine-enhanced DLI) [89]. In addition, leukemia-specific T cells (mLSTs) have recently been tested in an ongoing interventional phase I clinical trial (NCT02494167) [90]. Donor T cells from different donors and reactive to multiple LAAs (PRAME, WT1, Survivin and NY-ESO-1) were selectively activated and expanded [90]. In vitro, only leukemia cells were attacked by these selected T cells. The administration was not associated with extensive GvHD, and the efficacy of these products seems promising [90]. In a still-recruiting phase I/II trial, NEXI-001 T-Cells with the targets PRAME/WT1/Cyclin A1 are infused after alloHSCT in AML or MDS patients with the goal of enhanced GvL effect without increasing the incidence of GvHD, cytokine release syndrome or neurotoxicity (NCT04284228; [91]). However, further clinical research is needed.

## 5. Immunomodulation in AML

AML is a disease of older patients; many of these patients are not strong enough for intensive treatment [92]. A successful approach in this setting is to use DNA-hypomethylating (HMAs) agents, such as azacitidine (AZA) or decitabine (DAC). DNA-hypomethylating agents cause differentiation and apoptosis of AML blasts. The side effects are quite tolerable even in older and unfit patients. Preclinical trials demonstrated that HMAs cause a significant enrichment for immunomodulatory pathways in different cancers cells [93].

Indeed, HMAs have been reported to upregulate the expression of several testicular cancer antigens such as NY-ESO-1 and MAGE. However, while on the one hand HMAs stimulate the immune response against tumor antigens, on the other hand inhibitory immune checkpoint molecules are upregulated [94]. This mechanism may crucially contribute to the development of drug resistance [94]. In summary, their immunomodulatory properties make them an interesting backbone for combination therapies [95]. Especially since combination therapies might comprise the possibility to bypass drug resistance mechanisms. In various clinical trials, AZA and DAC have been shown to be highly effective drugs in combination therapies due to their good safety profile. [94]. These phase I/II trials with anti-PD-1/PD-L1 antibodies in combination with HMAs have shown encouraging and durable response rates, however only few patients were included, the toxicity was high and there are no randomized studies (NCT02397720) [94,96]. The combination of HMAs plays an important role in the treatment of AML. Especially the combination with venetoclax has shown promising response rates [97]. Combination trials of ivosidenib or enasidenib with AZA in relapsed or refractory AML (NCT03683433) as well as in first-line therapy of *IDH*mut AML with enasidenib (AML005-trial) or ivosidenib (AGIL-trial) in combination with AZA (NCT02677922) are currently ongoing [97,98]. Even in the posttransplant setting several studies showed the benefit of the combination of DLIs and AZA, further randomized trials are, up to date, still lacking [95]. Additionally, the therapy with DLIs and AZA can be enhanced with venetoclax in the posttransplant setting [95]. Another combination partner with HMAs in the posttransplant setting is lenalidomide [95]. While lenalidomide as a single agent caused high rates of GvHD, there were acceptable side effects and good response rates when combined with HMAs [95].

To address the common issue of relapse after induction therapy, a phase III trial is currently investigating maintenance therapy with cc-486, an oral AZA formulation [99]. The median overall survival as well as the median relapse-free survival was significantly improved in the cc-486 group. In addition, the quality of life was maintained in the cc-486 group [99].

## 6. Non-Immunogenic Targeted Therapies in AML

This part of the review is not the focus of this work. Nevertheless, for the sake of completeness, we would like to make a brief excursion into this topic.

**FLT3 inhibitors:***FLT3* mutations are activators of the signal transduction cascade by PI3K/AKT/mTOR, RAS/MAPK and STAT5 [53]. By this mechanism, FLT3 inhibitors develop cytotoxic potential to cell lines, and primary AML cells accommodate *FLT3* mutations [100]. First-generation broad-spectrum tyrosine kinase inhibitors (TKIs) such as midostaurin, as well as sorafenib and lestaurtinib, provide limited and temporary antileukemic activity as single agents [40,53,101]. However, midostaurin substantially improved OS and event-free survival compared to placebo in combination with standard chemotherapy in patients with *FLT3* mutations [53,56,102]. In the relapsed setting, gilteritinib was approved for use in *FLT3*-mutant AML by the FDA in late 2018 [40,53,101,103]. In older, unfit patients, another strategy is the use of FLT3 inhibitors in combination with hypomethylating agents, which has yielded promising results; however, in a phase III trial with Gilteritinib in combination with AZA, the OS was not improved compared to AZA alone (NCT02752035; [104]) [40,101]. Even in the after-alloHSCT setting as maintenance therapy, e.g., sorafenib translated in an improvement of OS (Sormain-trial) [40,101,105]. The recently developed FLT3 inhibitor quizartinib significantly improved OS in patients with relapsed/refractory AML compared with physician’s choice [40].

A new aspect is the use of FLT3 inhibitors as maintenance therapies after alloHSCT [53]. For example, in a phase III study, gilteritinib was investigated in the post-transplant phase (ClinicalTrials.gov identifier: NCT02997202) [40].

However, an issue in the personal tailored medicine is the development of resistance because of diverse escape mechanisms [101,106]. One possible escape mechanism is the increment of FLT3 ligands after cytotoxic treatment, which competes with the FLT3-Inhibitors [40].

**IDH-inhibitors.** Another target structure, feasible for targeted therapy is **isocitrate dehydrogenase 1 and 2** (**IDH1/2**) [40,53]. IDH1/2 are NADP+-dependent enzymes that catalyze the oxidative decarboxylation of isocitrate to α-ketoglutarate (α-KG) [40,53]. *IDH1R132* mutations are found in 5–10% of AML patients and are associated with clonal hematopoiesis of indeterminate potential (CHIP); an incident in the development of leukemia [53,107]. *IDH1* mutations are often associated with *NPM1* mutations [53,107].

The recently developed ivosidenib or enasidenib, IDH 1/2 inhibitors, are potent in *mIDH1/2* relapsed or refractory AML [108]. Further studies assess the safety and efficacy of ivosidenib or enasidenib in combination with chemotherapy in *mIDH1/2* AML [108]. The safety characterization of both IDH-inhibitors in combination with chemotherapy was comparable to those of chemotherapy alone [108]. In 39% (ivosidenib) and 23% (enasidenib) of patients, the *IDH1/2* mutation was no longer detectable [108]. Both IDH inhibitors were well tolerated [108].

**Glasdegib:** in combination with low-dose cytarabine, the hedgehog inhibitor **Glasdegib** demonstrated significant improved survival in elderly or unfit patients [109]. Recently, this combination was FDA approved in elderly patients with untreated AML [97]. Furthermore, in the BRIGHT AML 1019 (NCT03416179) study, a double-blind, randomized phase III trial, glasdegib was orally administered in patients with intensive chemotherapy and in the non-intensive treatment group in combination with AZA [109]. The OS survival in patients treated with glasdegib in combination with intensive chemotherapy in comparison to historical data seems to be promising [109].

**BCL-2 inhibitor:** the expression of **BCL-2 (B-cell lymphoma 2)** is elevated in AML blasts [53]. Even though BCL-2 is not a prognostic predictor in AML patients, the introduction of ABT-199 a BCL-2 inhibitor exemplified the therapeutic options [53]. In ex vivo and xenotransplant models, *IDH1*- and *IDH2*-mutant human AML cells were highly sensitive to BCL-2 inhibition [110]. These data suggest that *IDH1/2*-mutated AMLs are eligible for BCL-2 inhibition [110]. However, in targeted therapies, resistance mechanisms are a crucial aspect to consider. To circumvent resistance, the combination of two novel agents, FLT3 and BCL-2 inhibitors, has shown promising efficacy [53]. In addition to these encouraging results, incomplete hematological recovery was observed, which could indicate potentially increase toxicity to non-malignant hematopoietic progenitor cells [53]. Elderly patients with acute myeloid leukemia have an unfavorable prognosis, even after treatment with a hypomethylating agent [111]. Therefore, the VIALE-A study was initiated to investigate the efficacy and safety of the azacitidine–venetoclax combination compared to a control regimen of azacitidine and placebo in previously untreated patients with AML who were not eligible for intensive induction therapy [111]. The combination of azacitidine plus venetoclax was an effective treatment regimen resulting in a significant improvement in the frequency of complete remissions and overall survival, and it became a standard treatment for patients with non-intensive treatment [111].

Even for younger patients in the first line therapy or in the relapsed refractory AML setting as salvage therapy (FLA-V-IDA), the combination of venetoclax with intensive treatment has shown promising results [112,113]. In addition, further combinations such as gilteritinib with venetoclax, in refractory/relapsed *FLT3*mut AML (NCT03625505), are under investigation [97]. It remains exciting to see the further development of the possible drug combination in AML therapy.

**APR-246 (Eprenetapopt):** a promising new agent is **APR-246,** which stimulates the transcriptional activity of p53 mutants and even can induce p53-independent apoptosis [114]. Therefore, APR-246 may be even able to overcome chemo-resistance [115]. However, a phase III trial with Eprenetapopt in combination with azacitidine in patients with *TP53*-mutant MDS missed the primary end point of CR (NCT03745716). Therefore, further clinical trials are needed to gain knowledge about the efficacy and side effects both as a single agent and as a combination therapy.

In de novo or secondary AML, a **KMT2A (also known as MLL) rearrangement** is found in 1–2% of patients and in ~15% of patients with therapy-related AML [53]. There are more than 70 fusion partners identified while MLL, MLLT3 (9p21.3), MLLT4 (6q27), ELL (19p13.1) and MLLT10 (10p12.3) are the most frequent [53]. KMT2A rearrangement conveys a poor prognosis [40]. Mutant MLL proteins divert DOT1L to different targets [40]. A newly developed inhibitor of DOT1L, pinometostat (EPZ-5676), disrupts the leukemogenesis [40]. In phase I/II studies, pinometostat appeared safe [40]. A new investigational oral drug **KO-539** targets the menin-KMT2A protein interaction. In a phase I/IIa trial, KO-539 is orally administered in relapsed or refractory AML patients [116]. Up to date, there were no dose-limiting toxicities, and the medication was well-tolerated. The biological activity of the new drug seems promising [116]. Further results are expected soon.

In the AUGMENT-101 phase I/II trial **SNDX-5613,** a menin-MLL small molecule inhibitor is administered in mixed-lineage leukemia rearranged (KMT2A) and *NPM1*mut-relapsed or -refractory acute leukemias (NCT04065399) [117]. The efficacy as well as the toxicity profile are encouraging, and SNDX-5613 may potentially lead to a better prognosis in AML with MLL rearrangement [117]. However, further clinical trials are needed. 

**Figure 2:**
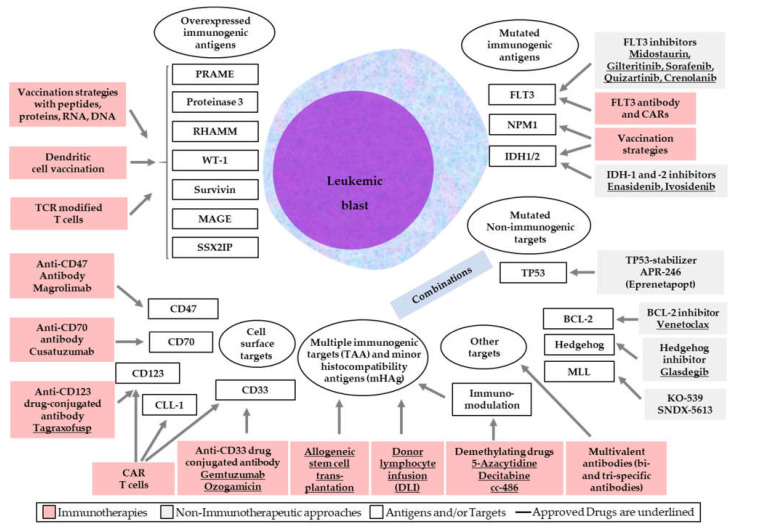
Increasing role of targeted and non-targeted immunotherapies (red) including approved (underlined) and non-approved drugs in the portfolio of AML treatment.

All immunotherapeutic approaches are displayed in Figure 2 (see also the Supplementary Materials).

## 7. Conclusions

New treatment strategies for AML patients have been developed in the past few years. While the median overall survival in AML patients has increased over time, the treatment of patients without response or relapse is still associated with a limited prognosis. In this setting, immunotherapy might be an option to prevent and treat relapse in AML patients. For example, alloHSCT and DLIs are standard clinical practice in high-risk AML patients; however, new immunotherapeutic approaches are becoming more and more popular and need to be further developed. AML is a heterogeneous, often oligoclonal disease, so the search for targets for immunotherapy in this disease entity is ongoing. Most efforts to discover antigens for immunotherapy have focused on proteins that are overexpressed in leukemia, or LAA.

Many of these have been extensively researched, validated and, in some cases, discarded as viable targets for immunotherapy. However, LAAs might trigger the elimination of leukemic blast by CTLs. In conclusion LAAs might be promising targets for an immunotherapeutic approach. Up to date, several important LAAs have been identified and used in vaccination trials, and further immunogenic treatments based on LAAs will follow. Nevertheless, it must be said that a protein target found in all AMLs and all subtypes may not exist or has yet to be determined [118].

Since the revolution of the AML therapy by cytarabine and daunorubicin (7 + 3), the AML therapy was homogenous for all different kinds of AML subgroups and patients. Due to next-generation sequencing (NGS) and genomic profiling, a higher level of comprehension was achieved.

Based on a better comprehension of the proliferation, apoptosis and immune escape mechanisms of cancer cells as well as specific vulnerabilities, immunogenic target structures could be identified and translated into targeted therapy approaches [60].

Owing to these approaches the AML therapy was improved and individualized progressively. Since 2017, nine new drugs were implemented in the treatment of AML [53]. First and foremost, the BCL-2 inhibitor venetoclax [119] but also, for example, the FLT3-inhibitor midostaurin and the IDH-inhibitors changed AML treatment significantly. New, already ongoing AML trials, such as the Biomarker-Based Treatment (BEAT) trial or further planned studies of the AML-SG, acknowledge the heterogeneity of AML and assign patients to therapies based on the molecular subtype.

However, besides the considerable improvement in the ongoing development of multiple molecular and immunological therapeutic targets, there are some substantial aspects to be addressed.

One aspect is the critical evaluation of the efficacy of the newly developed therapy. New therapeutic drugs are first tested and implemented in the treatment of patients with relapsed or refractory disease. In this setting, it is difficult to assess the efficacy of the therapy. First, relapsed or refractory AMLs are often associated with a high tumor burden; therefore, targeted therapy might not have enough therapeutic force to overcome a large tumor mass, even if, in a much less aggravated disease setting, the drugs would be highly effective. Second, the targeted therapy might not take effect in time due to the rapid growth of the leukemic cells, especially in the heavily pre-treated situation, which means falling behind the leukemic growth, while in a smoldering disease setting, this challenge would not be of significance.

Last but not least, there is the question of how to deal with resistance mechanisms in targeted therapy. Cancer cells are highly adaptable due to clonal evolution [120]. In the pathogenesis of the AML, hematopoietic stem/progenitor cells acquire somatic mutations over time, and this leads to uncontrolled expansion [121]. Therefore, over time, the further evolution of subclones leads to resistance mechanisms, immune escape and the further growth of leukemic cells [53].

The solution for these issues is to combine different targeted drugs, to reduce the tumor burden, control the rapid growth and prevent resistance mechanisms. This raises the question of which treatment concepts are most effective and how different treatment strategies can be combined and implemented in the clinical setting. Due to the countless possible combinations, this is a constant and time-consuming undertaking. All these aspects have to be taken in consideration, and further studies are urgently needed. However, even if only a small fraction of this newly developed drugs will be paradigm changing, the tailored treatment is an ongoing and further developing progress.

In conclusion, there is a high potential for immunotherapeutic therapies to address the barriers in AML treatment mentioned above. Especially, the combination of conventional treatment with further innovative immunogenic strategies might result in a substantial improvement in the therapy and survival of AML patients.

## Supplementary Materials

The following supporting information can be downloaded at https://www.mdpi.com/article/10.3390/ijms23063304/s1.
