# Supplementary material for: Increasing Role of Targeted Immunotherapies in the Treatment of AML
Source: Int J Mol Sci. 2022 Mar 18;23(6):3304. doi: 10.3390/ijms23063304 (PMC8953556; doi:10.3390/ijms23063304)
Supplement: Supplementary file 1 [file ijms-23-03304-s001.zip › ijms-1580488-supplementary.pdf]

Treatment of AML						
**Target sturcture**	**drug/procedure**	**advantage**	**disadvantage**	**clinical trials**	**status**	**NCT/EudraCT/ISRCTN/Reference**
BCL-2	Venetoclax in combination with azacitidine	Feasible for patients with comorbidites and poor performance status	Adverse events of grade ≥ 3; thrombocytopenia, neutropenia and febrile neutropenia	Phase III	Active, not recruiting	VIALE-A; NCT02993523; DiNardo et al. N Engl J Med, 2020
CD123	CART123 cells; cyclophosphamide; fludarabine	In relapsed/refractory AML; activity of CART demonstrated by the CRS;	Not yet standard therapy for AML;' no significiant clinical response; transient CAR T cell detection in vivo	Phase I	Active, not recruiting	NCT03766126; Isidori et al. Front Oncol, 2021
CD123	Tagraxofusp-erzs (SL-401)	In relapsed/refractory CD123+ AML or Blastic Plasmacytoid Dendritic Cell Neoplasm Immunophenotype-like (BPDCN-IPh-like) AML	Ongoing trial; no results published	Phase II	Recruiting	NCT04342962
CD33	Gemtuzumab-Ozogamicin in combination with standard chemotherapy	Untreated de novo AML; improved outcome	Persistent thrombocytopenia	Phase III	Completed	EudraCT 2007-002933-36; Castaigne et al. Lancet, 2012
CD33	Gemtuzumab-Ozogamicin in combination with standard chemotherapy	NPM1-mutated AML; significantly fewer relapses occurred in the GO compared with the standard arm	EFS was not met, mainly as a result of higher early death rate in the GO arm	Phase III	Completed	AMLSG 09-09; NCT00893399; Schlenk et al. J Clin Oncol, 2020
CD47	Magrolimab in combination with azacitidine and venetoclax	In relapsed/refractory AML; high CR/CRi (94%) rates	Anemia should be monitored	Phase Ib/II	Recruiting	NCT04435691; Daver et al. ASH, 2021
CD70	Cusatuzumab (ARGX-110) in combination with azacitidine	Treatment reduced LSCs and triggered gene signatures related to myeloid differentiation and apoptosis	Rare infusion-related reactions; hematological toxicities related to azacitidine	Phase I/II	Active, not recruiting	NCT03030612; Riether et al. Nat Med, 2020
CLEC12A/CLL1	MCLA-117 bispecific antibody	In relapsed/refractory AML and newly diagnosed untreated AML patients with high risk cytogenetics; high expression in AML/not in normal HSC	The exact physiological function of CLL-1 is not understood yet; may lead to autoimmune dieases; trial is still ongoing	Phase I	Active, not recruiting	NCT03038230; Ma et al. Journal of Hematology & Oncology, 2019
DNA methyltransferase	Oral azacitidine (cc-486) maintenance	Significantly improved overall and relapse-free survival in patients with AML in remission after chemotherapy	Controllable side effects were mainly gastrointestinal adverse events and neutropenia	Phase III	Active, not recruiting	NCT01757535; Wei et al. N Engl J Med, 2020
Exportin1 (XPO1)	Selinexor (KPT-330)	Patients ≥60 years with relapsed/refractory AML	Increased incidence of adverse events ≥3 (thrombocyto-penia, febrile neutropenia, anemia, hyponatremia)	Phase II	Completed	NCT02088541; Sweet et al. Leuk Lymphoma, 2021
FLT3	Midostaurin in combination with standard chemotherapy	Addition of midostaurin significantly improved overall (P=0.009) and event-free survival (P=0.002)	The rate of grade ≥3 anemia was higher in the midostaurin group (P=0.03), as was the rate of rash (P=0.008)	Phase III	Active, not recruiting	NCT00651261; CALGB 10603 (RATIFY); Stone et al. N Engl J Med, 2017
FLT3	4G8-SDIEM (Fc-optimized antibody FLYSYN) monotherapy	Safe and well tolerated in patients with molecular MRD	Unspecific adverse events (fatigue, flu like symptoms, musculoskeletal symptoms and laboratory abnormalities)	Phase I/II	Completed	NCT02789254; Heitmann et al. Blood, 2020
Hedgehog pathway	Glasdegib in combination with azacitidine or standard chemotherapy	Significant antitumor efficacy in vivo and clinical activity in Phase I and Ib/II trials in patients with untreated AML	Side effects like alopecia, muscle spasms and dysgeusia	Phase III	Completed	NCT03416179; BRIGHT AML 1019; Cortes et al. Future Oncol, 2019
Histon-Deacetylase (HDAC)	Azacitidine in combination with vorinostat	There was significant antileukemia activity and vorinostat effectively inhibited HDAC activity in peripheral blood and bone marrow blasts	Dose-limiting toxicities included anorexia, dehydration, diarrhea, and fatigue	Phase II	Completed	NCT01617226; RAvVA; Garcia-Manero et al. Blood, 2008
IDH-2	Azacitidine in combination with enasidenib	Well-tolerated and effective in elderly pts with IDH-2mut untreated AML as well as pts with refractory/relapsed AML	Adverse events were IDH differentiation syndrome (8%) and indirect hyperbilirubinemia (35%)	Phase II	Unknown	NCT03683433; Venugopal et al. Blood Cancer J, 2022
MAGE	NPM1-peptide vaccine against NY-ESO-1, PRAME, MAGE-A3, WT-1 in combi nation with azacitidine	Five high risk MDS patients were treated; no serious adverse events	The trial was terminated early as there was no sign of clinical benefit or immunological response	Phase I	Completed	NCT02750995; Holmber-Thydén et al. ASH, 2020
Menin-MLL (KMT2A)	KO-539	Orally administered; in relapsed/refractory AML	Further data is needed	Phase I/II	Recruiting	NCT04067336; KOMET-001; Wang et al. ASH, 2020
Menin-MLL (KMT2A)	SNDX-5613	Orally administered; in relapsed/refractory leukemia	Adverse events were QT prolongation, anemia, and differentiation syndrome	Phase I/II	Recruiting	NCT04065399; AUGMENT-101; Tucker et al. Targeted Oncology, 2021
PRAME/WT1/Cyclin A1	Infusion of NEXI-001 T Cells after alloHSCT	Enhance GvL effect without increasing the incidence of GvHD, cytokine release syndrome or neurotoxicity	Donor-derived adoptive cellular therapy product. Time mangement, donor consent and production limitations	Phase I/II	Recruiting	NCT04284228; Malki et al. ASCO, 2021
Proteinase 3/PR1	PR1 leukemia peptide vaccine in combination with Montanide ISA-51	Reduction of disease activity in 22% (12/53) in patient with CML, AML or MDS	Not feasible in patients with high leukemic burden; Vaccine HLA-A2 restricted	Phase I/II	Completed	NCT00004918; Qazilbash et al. Leukemia, 2017
RHAMM	1. RHAMM-R3 vaccine<br>2. High-dose RHAMM-R3 vaccine	Increase of R3-specific CD8+ T cells and reduction of blasts in bone marrow	Not feasible in patients with high leukemic burden; Not only AML patients recruited; Vaccine HLA-A2 restricted	Phase I/II	Completed	ISRCTN32763606 and EudraCT as 2005-001706-37; Schmitt et al. Blood, 2008; Greiner et al. Haematol., 2010
SSX2IP	Up to date there is no drug available	SSX2IP expression at disease presentation predicts good survival with no detectable cytogenetic rearrangements		No clinical trial available		Davis et al. Cancers (Basel), 2020
Survivin	YM155 survivin suppressant	To cancer biologists, survivin is one of the most tumour-specific molecules	Further research is needed	No clinical trial available		Nakahara et al. Cancer res, 2007
TP53	[illegible] stores wild-type p53 functions in TP53-mutant cells	In combination with azacitidine as maintenance therapy after alloHSCT	Elderly and patients with renal failure may be vulnerable for transient neurologic side effects	Phase II	Completed	NCT03931291; Sallman et al. Journal of Clinical Oncology, 2021
WT-1	WT1 126-134 peptide vaccine	WT1-expressing AML/ MDS without curative treatment option	Low patient number; Vaccine HLA-A2 restricted	Unknown	Unknown	NCT unkown; Keilholz et al. Blood, 2009
WT-1	WT1 peptide vaccine (galinpepimut-S)	Stimulation of a specific immune response, and survival in excess of 5 years	Clinical efficacy needs to be addressed in a larger trial with more homogeneous patient population	Phase II	Completed	NCT01266083; Maslak et al. Blood Adv., 2018
WT-1	WT1 mRNA-electroporated Dendritic Cell Vaccination	WT1-targeted DC vaccination can elicit anti-leukemia T cell immunity in patients with AML at very high risk of relapse	Not feasible in patients with high leukemic burden; Not only AML patients recruited;	Phase II	Unknown	NCT00965224; Anguille et al. Blood, 2017
